# The impact of COVID-19 pandemic on air particulate matter exposure and heart attacks: a 5-year retrospective cohort study in Taiwan (2017–2021)

**DOI:** 10.3389/fpubh.2024.1321129

**Published:** 2024-02-27

**Authors:** Chih-Chien Yen, Po-Jen Hsiao, Chi-Ming Chu, Ping-Ling Chen

**Affiliations:** ^1^Department of Surgery, Division of Cardiovascular Surgery, Taoyuan Armed Forces General Hospital, Taoyuan, Taiwan; ^2^Department of Surgery, Division of Cardiovascular Surgery, Tri-Service General Hospital, National Defense Medical Center, Taipei, Taiwan; ^3^Graduate Institute of Injury Prevention and Control, College of Public Health, Taipei Medical University, Taipei, Taiwan; ^4^Department of Internal Medicine, Division of Nephrology, Taoyuan Armed Forces General Hospital, Taoyuan, Taiwan; ^5^Department of Internal Medicine, Division of Nephrology, Tri-Service General Hospital, National Defense Medical Center, Taipei, Taiwan; ^6^Department of Life Sciences, National Central University, Taoyuan, Taiwan; ^7^School of Public Health, National Defense Medical Center, Taipei, Taiwan; ^8^Graduate Institute of Life Sciences, National Defense Medical Center, Taipei, Taiwan; ^9^Graduate Institute of Medical Sciences, National Defense Medical Center, Taipei, Taiwan; ^10^Department of Public Health, School of Public Health, China Medical University, Taichung, Taiwan

**Keywords:** COVID-19 pandemic, particulate matter, heart attacks, acute myocardial infarction, acute decompensated heart failure

## Abstract

**Background:**

Heart attacks including acute ST-segment elevation myocardial infarction (STEMI) and acute decompensated heart failure (ADHF) caused from the particulate matter (PM) and air pollutant exposures are positively associated with regional air pollution severity and individual exposure. The exceptional coronavirus disease epidemic of 2019 (COVID-19) may enhance the air conditions in areas under COVID-19 pandemic. We sought to study the impact of COVID-19 pandemic on air particulate matter (PM) exposure and heart attacks in Taiwan.

**Methods:**

This retrospective cohort study was conducted in one teaching hospital in Taichung, Taiwan. We examined emergency patients diagnosed with acute STEMI and ADHF from January 1, 2017, to March 31, 2020, (i.e., before the COVID-19 pandemic) and from April 1, 2020, to December 31, 2021, (after the COVID-19 pandemic). The effects of particulate matter with a diameter of less than 2.5 micrometers (PM_2.5_) and PM_10_ as well as temperature and humidity on environmental air pollutants were recorded. The analysis was performed with a unidirectional case-crossover research design and a conditional logistic regression model.

**Results:**

Both PM_2.5_ and PM_10_ levels had a positive association with the risk of acute STEMI before the COVID-19 pandemic (PM_2.5_ adjusted odds ratio (OR): 1.016, 95% confidence interval (CI): 1.003–1.032 and PM_10_ adjusted OR: 1.009, 95% CI: 1.001–1.018) and ADHF (PM_2.5_ adjusted OR: 1.046, 95% CI: 1.034–1.067 and PM_10_ adjusted OR: 1.023, 95% CI: 1.027–1.047). Moreover, the results demonstrated that PM_2.5_ and PM_10_ were not associated with the risk of acute STEMI or ADHF after the COVID-19 pandemic. Reduction in PM_2.5_ and PM_10_ levels after the COVID-19 pandemic were noted. Hospital admissions for acute STEMI (7.4 and 5.8/per month) and ADHF (9.7 and 8.2/per month) also decreased (21.6 and 15.5%) after the COVID-19 pandemic.

**Conclusion:**

In Taiwan, paradoxical reductions in PM_2.5_ and PM_10_ levels during the COVID-19 pandemic may decrease the number of hospital admissions for acute STEMI and ADHF. As the COVID-19 pandemic eases, the condition of air pollution may gradually become worse again. The governments should formulate better policies to improve the health of the public and the quality of the air.

## 1 Introduction

The greatest environmental risk to human health is air pollution (1, 2). Exposure to air pollution can increase oxidative stress, which can cause immunological abnormalities and inflammatory responses that induce cardiovascular disease (3–6). Good air quality is crucial to ensure human health and a liveable environment. Recent years have seen an increase in health problems due to increased air pollution from heating, traffic, and industry, especially in cities. The World Health Organization (WHO) has highlighted the link between lower levels of air pollution and better cardiovascular health in both the long and short term. Heart attacks usually develop when the blood flow of the heart is severely blocked or acute heart failure. Scholars have also found that minimizing air pollution significantly reduces cardiovascular disease and life expectancy loss (7–9).

Coronavirus disease 2019 (COVID-19) is an infectious disease caused by severe acute respiratory syndrome coronavirus type 2 (SARS-CoV-2). Furthermore, the COVID-19 pandemic is a major health hazard event that has led the government to strictly enact certain transmission blocking policies. At the same time, the COVID-19 pandemic has also led to an improvement in global air pollution. On March 11, 2020, the WHO declared COVID-19 a pandemic. In response, many governments instituted pandemic policies, which included strategies such as social distancing, mask wearing, travel restrictions, business restrictions and closures, workplace hazard controls, isolation, testing systems, and contact tracing. This also affected the severity of global air pollution and real-time individual exposure to air pollution (10, 11). Compared with most countries, Taiwan adopted a strict COVID-19 pandemic policy since April 2020 to February 2022 and thus only had an incredibly small number of confirmed COVID-19 patients. Cardiovascular disease prevention has always been an important health promotion policy worldwide. Alongside traditional personal risk factors, external environmental components have also increasingly attracted attention in recent years. The reduction in air pollution is an important issue.

The aim of this study was to determine how certain COVID-19 pandemic policies affected the reduction in air pollution severity, as well as individual exposures to air pollution. Furthermore, an investigation was conducted regarding whether such COVID-19 pandemic policies influenced the positive association between air pollution exposure and the incidence of heart attacks, including acute ST-segment elevation myocardial infarction (STEMI) and acute decompensated heart failure (ADHF).

## 2 Materials and methods

### 2.1 Data source and study population

This study was conducted in Taichung Armed Forces General Hospital, a 450-bed, regional, acute and critical care hospital in central Taiwan, which is located in an area with severe air pollution. This study examined emergency patients diagnosed with acute STEMI (i.e., an abnormal electrocardiogram, which is an elevated number of myocardial enzymes confirmed via cardiac catheterization) and ADHF (i.e., abnormal physical examination with abnormal echocardiography findings and an elevated NT-ProBNP level) during the periods from January 1, 2017, to March 31, 2020, (i.e., before the COVID-19 pandemic) and April 1, 2020, to December 31, 2021 (after the COVID-19 pandemic). This research excluded residents or workers who had spent less than 1 year in the area. People with confirmed COVID-19 infection were also excluded from this study. Based on emergency department and inpatient medical records, information such as the date of illness onset, sex, age, body mass index, hypertension, dyslipidaemia, diabetes mellitus, smoking, drinking, and heart disease history was collected. In addition, smoking and drinking habits were defined according to the recommendations of Taiwan’s Health Promotion Administration.

**Figure 1:**
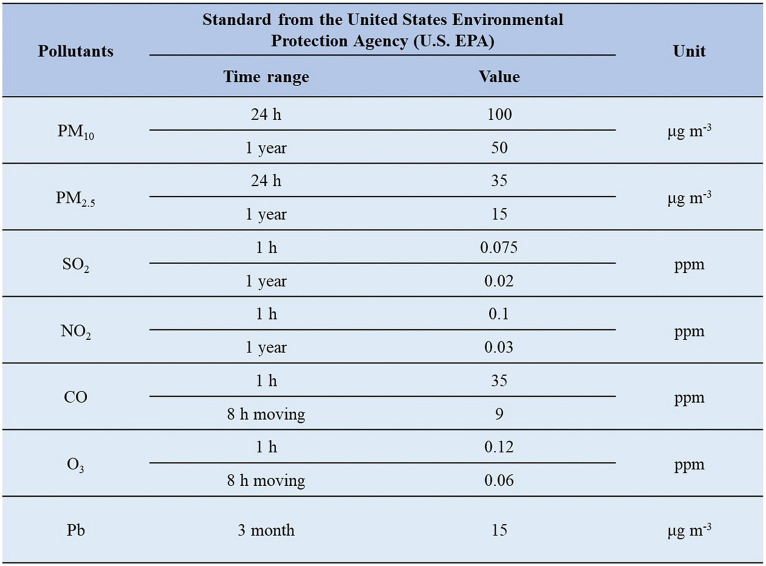
Taiwan’s air quality index (AQI) is reflected by the amounts of O_3_, PM_2.5_, PM_10_, CO, SO_2_, and NO_2_ in the air.

Data on environmental air pollutants were derived from the Executive Yuan’s Air Quality Monitoring Station of the Environmental Protection Agency. Each site analyses air pollutants hourly and reports the average 24-h daily concentrations of pollutants such as particulate matter with a diameter of less than 2.5 micrometers (PM_2.5_), PM_10_, ozone (O_3_), sulfur dioxide (SO_2_), nitrogen dioxide (NO_2_), and nitric oxide (CO) as well as temperature and humidity. The air quality standard of each air pollutant is stipulated as the following: Figure 1. Taiwan’s air quality index (AQI) is reflected by the amounts of O_3_, PM_2.5_, PM_10_, CO, SO_2_, and NO_2_ in the air.^1^ The index is divided into six levels: good (green), average (yellow), unhealthy for sensitive groups (orange), unhealthy for everyone (red), very unhealthy (purple), and harmful (brown). According to the recommendations of Taiwan’s AQI, a value greater than 100 (orange) indicates poor air quality. The following regulations are considered to be met if the concentration of fine particulate matter in total quantity control zones and air pollution control zones is in compliance with them: (a) For general monitoring stations, list the valid 24-h value for each year within said zones in ascending order from lowest to highest. Calculate the average value over three consecutive years using the value that corresponds to the 98th cumulative percentage. The result should be less than the fine particulate matter 24-h value in the air quality standards when this value is averaged with the average values of other stations; Get the average of a station’s average values over three consecutive years using general monitoring stations. (b) Then, get the average of all the stations in the zone. The calculated value ought to be lower than the fine particulate matter standards’ annual average value. The general monitoring stations mentioned above are those that the central competent authority installed or approved. If less than 75% of the values a monitoring station monitors in a year are represented by its valid values, the data from that station will not be taken into account. The central competent authority must decide whether substances other than fine particulate matter are in compliance with air quality standards.

### 2.2 Statistical analysis

We investigated the relationships between exposure to air pollution and heart attacks, including STEMI and ADHF, using a unidirectional case-crossover research design (before the COVID-19 pandemic 1:1 matching and after the COVID-19 pandemic 1:2 matching). The ambient air pollutants concentration was calculated based on the average concentration on the current day and previous day (lag01). Two and four weeks prior to the date of the patient’s illness onset, the levels of ambient air pollutants were compared with each other. The 95% confidence intervals (CIs) and adjusted odds ratios (adjusted ORs) were calculated using the conditional logistic regression model. Moreover, the daily temperature and relative humidity were considered weather control factors.

In this study, a *t* test was performed to compare continuous variables, presented as the means ± standard deviations. In addition, the χ2 test and Student’s *t* test were used to compare categorical variables between the groups. The statistical significance level was set at *p* < 0.05. Furthermore, SPSS version 22 (Chicago, IL, United States) was used for data analysis.

## 3 Results

According to the emergency department and inpatient medical records, 289 and 122 acute STEMI patients and 378 and 172 ADHF patients from Taichung Hospital during the periods of January 1, 2017, to March 31, 2020 (before the COVID-19 pandemic) and from April 1, 2020, to December 31, 2021 (after the COVID-19 pandemic) were enrolled in this study.

**Figure 2:**
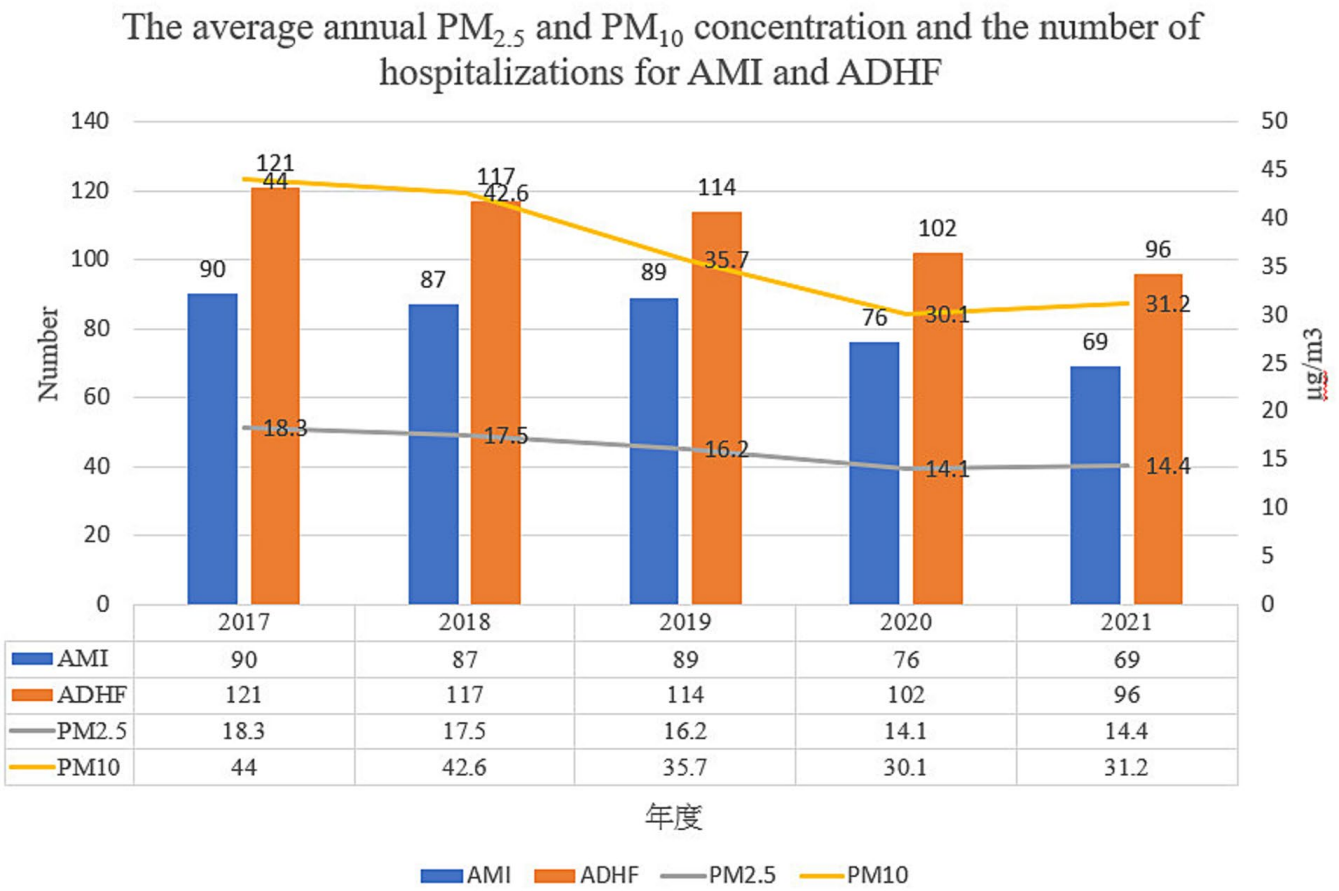
The average annual PM_2.5_ and PM_10_ concentration and the number of hospitalizations for STEMI and ADHF.

The demographic and medical information of the included acute STEMI patients before and after the COVID-19 pandemic is shown in Table 1 for the two time periods. Prior to the COVID-19 pandemic, patients were mostly male (68.86%) and the mean age at acute STEMI onset was 64 years (64.13 11.71). The patients’ average body mass index was overweight (27.71 ± 4.89). Moreover, the primary risk factors were hypertension and smoking. After the COVID-19 pandemic, the mean age regarding acute STEMI onset was 67 years (67.35 ± 13.09), and most patients were male (68.03%). Furthermore, their average body mass index was also overweight (28.53 ± 5.01). Similar to the before COVID-19 pandemic group, the typical primary risk factors were hypertension and smoking. No statistically significant variations were observed concerning demographic or medically related factors, except with respect to smoking and drinking, across the cases collected from Taichung Hospital in both periods. After the COVID-19 pandemic, rates of smoking and drinking were higher than before the COVID-19 pandemic. Figure 2 show the average annual PM_2.5_ and PM_10_ concentration and the number of hospitalizations for STEMI and ADHF. After the COVID-19 pandemic, the average annual PM_2.5_ and PM_10_ concentration were decrease and the number of hospitalizations for STEMI and ADHF were also decrease.

**Table 1:** Acute ST-segment elevation myocardial infarction (STEMI) patients’ demographic and medical variables before and after the COVID-19 pandemic.

Acute STEMI	Before		After		
	Number	Percentage	Number	Percentage	*p*
**Total (per month)**	289 (7.4/M)	100	122 (5.8/M)	100	0.018
**Gender**					0.836
Male	199	68.86	83	68.03	
Female	90	31.14	39	31.97	
**Age**	64.13 ± 11.71		67.35 ± 13.09		0.279
Age group (year)					
<45	53	18.34	15	12.30	
45–65	114	39.45	46	37.70	
>65	122	42.21	61	50.00	
**BMI (kg/m** ^**2**^ **)**	27.71 ± 4.89		28.53 ± 5.01		0.687
<18.5	8	2.77	3	2.46	
18.5 to <25	101	34.95	39	31.97	
25 to <30	131	45.33	58	47.54	
≧30	49	16.95	22	18.03	
**Hypertension**					0.874
Yes	203	70.24	86	70.49	
No	86	29.76	36	29.51	
**Dyslipidemia**					0.551
Yes	101	34.95	40	32.79	
No	188	65.05	82	67.21	
**Diabetes mellitus**					0.419
Yes	93	32.18	43	35.25	
No	196	67.82	79	64.75	
**Smoking**					0.019
Yes	173	59.86	91	74.59	
No	116	40.14	31	25.41	
**Drinking**					0.026
Yes	99	34.26	59	48.36	
No	190	65.74	63	51.64	
**Heart disease**					0.169
Yes	72	24.91	34	27.87	
No	217	75.09	88	72.13	

The results of the patients before the COVID-19 pandemic revealed that exposure to ambient air pollutants PM_2.5_ (2-week interval adjusted OR: 1.016, 95% CI: 1.003–1.032) and PM_10_ (2-week interval adjusted OR: 1.009, 95% CI: 1.001–1.018) had a positive association with an increase in the risk of acute STEMI (Table 2). Moreover, the results from the patients after the COVID-19 pandemic showed that exposure to PM_2.5_ (2-week interval adjusted OR: 1.010, 95% CI: 0.998–1.019 and 4-week interval adjusted OR: 1.005, 95% CI: 0.977–1.019) and PM_10_ (2-week interval adjusted OR: 1.005, 95% CI: 0.983–1.023 and 4-week interval adjusted OR: 1.004, 95% CI: 0.981–1.024) was not associated with the risk of acute STEMI (Table 3). Furthermore, the study was grouped and stratified based on the patient’s demographic and medical variables such as gender grouping or age stratification and the analysis results were consistent with the main analysis result.

**Table 2:** Adjusted odds ratio of short-term exposure to PM_2.5_, PM_10_, and STEMI among patients in Taichung Hospital before the COVID-19 pandemic.

Conditional logistic regression model				
	Adjusted OR	95% CI	95% CI	*p*
PM_2.5_ (2 weeks before STEMI onset)	1.016	1.003	1.032	0.026
PM_10_ (2 weeks before STEMI onset)	1.009	1.001	1.018	0.033

Adjusted OR adjusted odds ratio: adjusted variables include gender, age, body mass index, hypertension, dyslipidemia, diabetes mellitus, smoking, drinking, heart disease history, daily temperature and relative humidity, CI confidence interval.

**Table 3:** Adjusted odds ratio of short-term exposure to PM_2.5_, PM_10_, and STEMI among patients in Taichung Hospital after the COVID-19 pandemic.

Conditional logistic regression model				
	Adjusted OR	95% CI	95% CI	*p*
PM_2.5_ (2 weeks before STEMI onset)	1.010	0.998	1.019	0.211
PM_2.5_ (4 weeks before STEMI onset)	1.005	0.977	1.031	0.773
PM_10_ (2 weeks before STEMI onset)	1.005	0.983	1.023	0.534
PM_10_ (4 weeks before STEMI onset)	1.004	0.981	1.024	0.601

Adjusted OR adjusted odds ratio: adjusted variables include gender, age, body mass index, hypertension, dyslipidemia, diabetes mellitus, smoking, drinking, heart disease history, daily temperature and relative humidity, CI confidence interval.

The demographic and medical information for the ADHF patients who were included for the time before and after the COVID-19 pandemic is shown in Table 4. Before the COVID-19 pandemic, the mean age at ADHF onset was 67 years (67.31 ± 16.61), with the majority of patients being female (62.96%). The patients’ average body mass index was overweight (27.17 ± 6.23). In addition, the primary risk factors were hypertension, dyslipidaemia, and a history of heart disease. After the COVID-19 pandemic, the mean age at ADHF onset was 69 years (69.19 ± 17.53), and the majority of patients were female (59.88%). Their average body mass index was also overweight (28.11 ± 4.49). Similar to the before COVID-19 pandemic group, the typical primary risk factors were hypertension, dyslipidaemia, and a history of heart disease. There were no statistically significant differences in terms of demographic or medical-related factors, except with respect to smoking and drinking, across the cases collected from Taichung Hospital in both periods. After the COVID-19 pandemic, smoking and drinking rates were found to be higher than those before the COVID-19 pandemic.

**Table 4:** Acute decompensated heart failure (ADHF) patients’ demographic and medical variables before and after the COVID-19 pandemic.

ADHF	Before		After		
	Number	Percentage	Number	Percentage	*p*
**Total (per month)**	378 (9.7/M)	100	172 (8.2/M)	100	0.011
**Gender**					0.492
Male	140	37.04	69	40.12	
Female	238	62.96	103	59.88	
**Age**	67.31 ± 16.61		69.19 ± 17.53		0.278
Age group (year)					
<45	23	6.09	14	8.14	
45–65	102	26.98	47	27.33	
>65	253	66.93	111	64.53	
**BMI (kg/m** ^**2**^ **)**	27.17 ± 6.23		28.89 ± 5.37		0.345
<18.5	8	2.12	3	1.75	
18.5 to <25	116	30.68	51	29.65	
25 to <30	239	63.23	109	63.37	
≧30	15	3.97	9	5.23	
**Hypertension**					0.112
Yes	226	59.79	112	65.12	
No	152	40.21	60	34.88	
**Dyslipidemia**					0.401
Yes	195	51.59	97	56.40	
no	183	48.41	75	43.60	
**Diabetes mellitus**					
Yes	179	47.35	83	48.26	
No	199	52.65	89	51.74	
**Smoking**					0.026
Yes	155	41.01	93	54.07	
No	223	58.99	79	45.93	
**Drinking**					0.034
Yes	95	25.13	67	38.95	
No	283	74.87	105	61.05	
**Heart disease**					0.531
Yes	306	80.95	143	83.14	
No	72	19.05	29	16.86	

The findings showed that prior to the COVID-19 pandemic, patient exposure to ambient air pollutants PM_2.5_ (2-week interval adjusted OR: 1.046, 95% CI: 1.034–1.067) and PM_10_ (2-week interval adjusted OR: 1.023, 95% CI: 1.017–1.047) was positively associated with an increased the risk of ADHF (Table 5). Moreover, the results showed that patient exposure to PM_2.5_ (2-week interval adjusted OR: 1.06, 95% CI: 0.986–1.025 and 4-week interval adjusted OR: 1.002, 95% CI: 0.978–1.026) and PM_10_ (2-week interval adjusted OR: 1.007, 95% CI: 0.991–1.012 and 4-week interval adjusted OR: 1.003, 95% CI: 0.981–1.023) was not associated with the risk of ADHF after the COVID-19 pandemic (Table 6). Furthermore, the study was grouped and stratified based on the patient’s demographic and medical variables such as gender grouping or age stratification and the analysis results were consistent with the main analysis result.

**Table 5:** Adjusted odds ratio of short-term exposure to PM_2.5_, PM_10_, and ADHF among patients in Taichung Hospital before the COVID-19 pandemic.

Conditional logistic regression model				
	Adjusted OR	95% CI	95% CI	*p*
PM_2.5_ (2 weeks before ADHF onset)	1.046	1.034	1.067	< 0.01
PM_10_ (2 weeks before ADHF onset)	1.023	1.017	1.047	< 0.01

Adjusted OR adjusted odds ratio: adjusted variables include gender, age, body mass index, hypertension, dyslipidemia, diabetes mellitus, smoking, drinking, heart disease history, daily temperature and relative humidity, CI confidence interval.

**Table 6:** Adjusted odds ratio of short-term exposure to PM_2.5_, PM_10_, and ADHF among patients in Taichung Hospital after the COVID-19 pandemic.

Conditional logistic regression model				
	Adjusted OR	95% CI	95% CI	*p*
PM_2.5_ (2 weeks before ADHF onset)	1.006	0.986	1.025	0.883
PM_2.5_ (4 weeks before ADHF onset)	1.002	0.978	1.026	0.975
PM_10_ (2 weeks before ADHF onset)	1.007	0.991	1.012	0.568
PM_10_ (4 weeks before ADHF onset)	1.003	0.981	1.023	0.817

Adjusted OR adjusted odds ratio: adjusted variables include gender, age, body mass index, hypertension, dyslipidemia, diabetes mellitus, smoking, drinking, heart disease history, daily temperature and relative humidity, CI confidence interval.

## 4 Discussion

**Figure 3:**
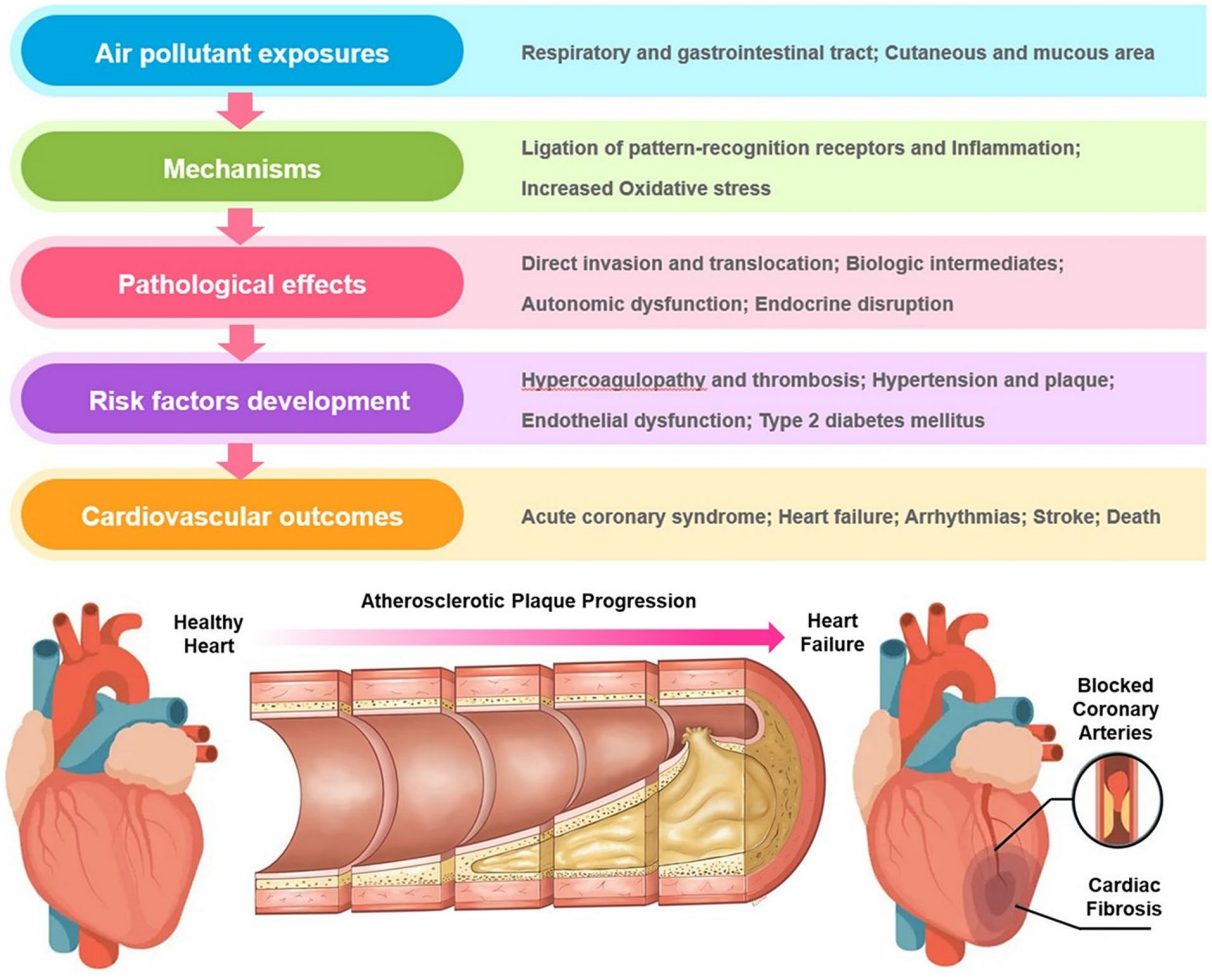
Cardiovascular effects and proposed mechanisms of exposure to air pollution.

Both PM_10_ and PM_2.5_ levels in the air are positively associated with cardiovascular diseases including acute STEMI and ADHF. Cardiovascular effects and proposed mechanisms of exposure to air pollution are shown in Figure 3 (11). Furthermore, this link varies according to the regional air pollution level. Areas with severe air pollution have a stronger association between acute STEMI and ADHF and exposure, but this correlation is negligible in places with low air pollution levels. (12, 13). Taiwan implemented strict COVID-19 lockdown procedures after the World Health Organization declared the virus to be a pandemic. Closures of industrial plants during the pandemic reduced industrial emissions, and the use of outdoor masks and working from home also decreased people’s exposure to air pollution. Restrictions against leaving one’s home also substantially diminished traffic emissions and reduced air pollution exposure. Air pollution severity after the COVID-19 pandemic was lower than that before the COVID-19 pandemic. Indeed, before the COVID-19 pandemic, the average PM_2.5_ concentration was 17.5 μg/m^3^, and the average PM_10_ concentration was 43.9 μg/m^3^, with 27% of days having a poor AQI. During the COVID-19 pandemic, the average PM_2.5_ concentration was 14.1 μg/m^3^, and the average PM_10_ concentration was 37.6 μg/m^3^, with only 14% of days having a poor AQI. The current results showed that before the COVID-19 pandemic, exposure to fine particulate matter and particulate matter pollutants had a positive association with an increase in the incidence rates of acute STEMI and ADHF; however, it must be noted that this positive correlation disappeared after COVID-19 pandemic policies were implemented. These results support the notion that some COVID-19 pandemic policies reduced the severity of air pollution and the positive correlation between exposure to fine particles and PM pollutants and acute STEMI and ADHF.

### 4.1 The accuracy of PM_2.5_ and PM_10_ measurements

As the Federal Equivalent Method (FEM) monitors, BAM-1020 (Met One Instruments Inc.) and TEOM-FDMS (1405-F or 1,405-DF, Thermo Fisher) serve as trustworthy near real-time monitors for compliance with the National Ambient Air Quality Standards and references for low-cost PM_2.5_ and PM_10_ sensor calibration (14–16). Consistency in PM_2.5_ and PM_10_ measurements is caused by differences between the FEM and FRM (Federal Reference Method) data, though. When the temperature, relative humidity, and PM_2.5_ and PM_10_ concentrations are known, the empirical equations can be used to correct the discrepancies between FEM and FRM values. The average bias for all stations and seasons for the FEM PM_2.5_ and PM_10_ is less than 10%. The FRM PM_2.5_ and PM_10_ can also be converted to the nearly “actual” PM_2.5_ and PM_10_ of the TEOM-FDMS (14–16). The empirical equations that have been derived should work in most parts of the world where the environmental conditions are similar.

### 4.2 The impact of COVID-19 pandemic on particulate matter air pollution in Taiwan

There is little information about the effects of the COVID-19 outbreak on regions that were not under lockdown, even though it greatly improved the air quality in the areas under lockdown provisions (17–19). A recent research framework is proposed to evaluate the long-term monthly spatiotemporal impact of COVID-19 on Taiwan air quality through geostatistical analysis, change detection analysis, and identification of nonattainment pollutant occurrence between the average mean air pollutant concentrations from 2018 to 2019 and 2020. Contrary to lockdown-imposed areas, COVID-19 started with negligible or deteriorating air quality conditions, but Taiwan experienced a delayed improvement after April. In comparison to 2018–2019, the annual mean concentrations of PM_10_, PM_2.5_, SO_2_, NO_2_, CO, and O_3_ were reduced by 24, 18, 15, 9.6, 7.4, and 1.3% in 2020, respectively. Over a 30 % reduction was observed in the overall frequency of nonattainment air pollutants (20).

### 4.3 The harmful effects of particulate matter on acute heart diseases before and after the COVID-19 pandemic

Mesnier et al. found that STEMI admissions in France underwent a marked decrease after the COVID-19 pandemic (21). This result is consistent with the findings in our study. During the COVID-19 pandemic, cardiovascular disease remained second among the top 10 causes of death in Taiwan, but a decrease was observed in the number of cases both before and after the COVID-19 pandemic. Before the COVID-19 pandemic, cases of acute STEMI averaged 7.4 per month, which dropped to 5.8 after the COVID-19 pandemic. Before the COVID-19 pandemic, cases of ADHF averaged 9.7 per month, which dropped to 8.2 after the COVID-19 pandemic (21). Acute STEMI and ADHF are life-threatening emergency diseases, and it is rare that medical treatment is refused even when the COVID-19 pandemic and the ensuing pandemic policies are ongoing.

Tables 1, 4 show an interesting finding that was observed after the COVID-19 pandemic, whereby there were significantly higher percentages of smoking and drinking among the study’s individuals. Additionally, during the COVID-19 pandemic, there was a non-significant positive association between air pollution and acute STEMI and ADHF due to reduced air pollution severity and exposure. This necessitates a higher focus on other traditional personal risk factors or indoor air pollution in relation to acute STEMI and ADHF.

**Figure 4:**
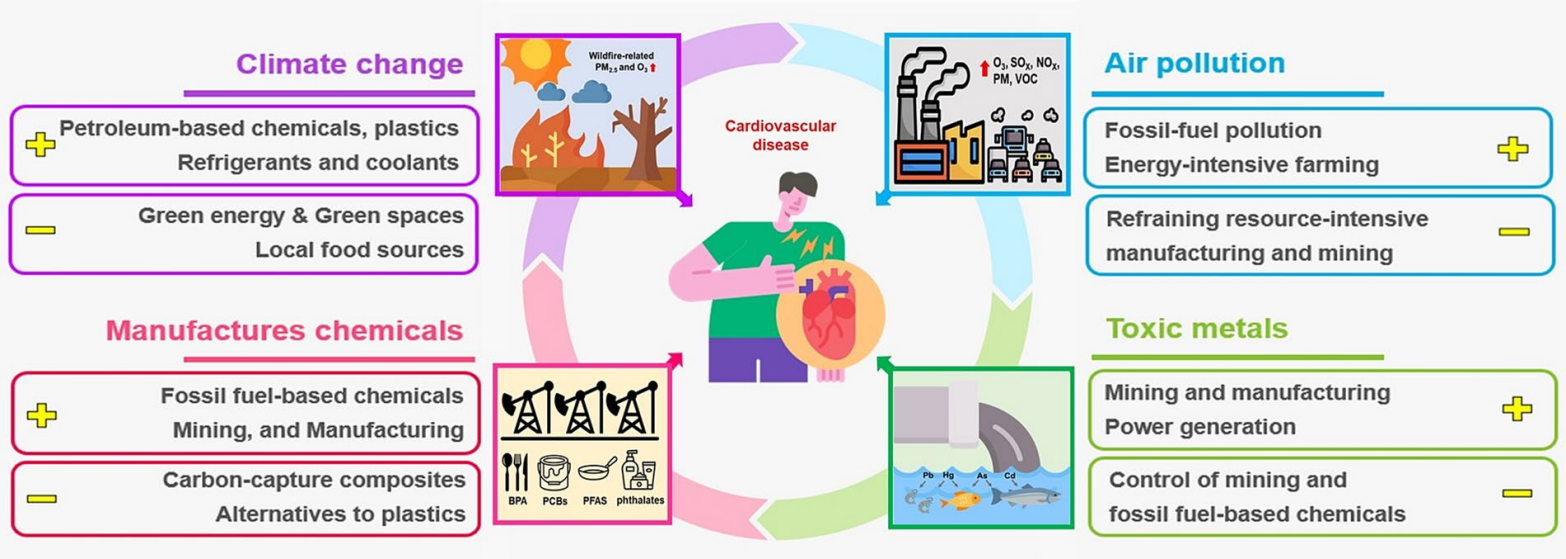
The harmful impact of worsening climate change and deteriorating air pollution to the cardiovascular health. Plus (+) and minus (−) signs designate potentiators and mitigators of pollution, respectively. As, arsenic; BPA, bisphenol A; Cd, cadmium; Hg, mercury; NOx, oxides of nitrogen; O3, ozone; Pb, lead; PCBs, polychlorinated biphenyls; PFAS, perfluoroalkyl substances; PM, particulate matter; PM_2.5_, PM that is less than 2.5 μm in aerodynamic-mass median diameter; Sox, oxides of sulfur; VOC, volatile organic compounds.

Air pollution refers to changes in the natural composition of the air due to various factors; foreign substances in the air take on solid, liquid, and gas forms, and their concentration and duration may be harmful to human health, life, ecological balance and commodities. While pollution can enter the atmosphere through the entry of foreign matter, it can also be impacted by geographical conditions, such as location and topographical features, as well as meteorological factors like temperature, pressure, precipitation, wind, humidity, and solar radiation. Interchangeability between worsening climate change and worsening air pollution could carry a significant threat to cardiovascular health (Figure 4). As air pollution severity is greatly affected by time, environmental, and climate factors, a single hospital setting rather than a wide area was utilized in this study.

A patient’s susceptibility to acute STEMI and ADHF is also influenced by their chronic health status, but these variables are frequently difficult to measure and change over time. An approach suggested by Maclure (22) to investigate the transient effects on acute event risk was used in this study, which employed a case-crossover study design. Most of the studies showed that the short-term exposure effect existed in the current day (lag0) and the previous day (lag1). The same result was obtained for previous study in Taiwan (12). Considering the impact of short-term exposure, the ambient air pollutants concentration used in this study was calculated based on the average concentration on the current day and previous day of case onset (lag01) (4, 12). However, a few studies indicate that sustained effects can occur within 1 week (23), so the study was originally designed to compare with 2 weeks ago to avoid sustained effects of air pollution exposure and reduce the differences between individuals and environments (13). The study result was positive correlation before the COVID-19 pandemic, but the correlation disappeared after the COVID-19 pandemic. Therefore, a 4 weeks ago design was added to improve the accuracy of the study result. We compared air pollution values in the event of acute STEMI and ADHF with those values both 2 and 4 weeks before the onset of patient illness. Intervals of 2 and 4 weeks also reduced weather factors and changes in the individual’s lifestyle and comorbidities. In addition, this study design helped to reduce the influence of individual differences and other confounding factors.

Recent research suggests that air pollution’s fine particulate matter and other particulate matter pollutants may worsen COVID-19’s severity and spread (24–27). Air pollution may also increase tissue inflammation and damage brought on by the virus by predisposing exposed people to COVID-19-related immunopathology (28). Certain studies have also found that COVID-19 mainly infects the respiratory system, but it also increases cardiovascular and neurological abnormalities. Additionally, patients suffering from cardiovascular and neurological diseases experience increased COVID-19 severity and mortality (29, 30). Due to the strict COVID-19 pandemic policies in Taiwan, only a few COVID-19 cases were confirmed from April 1, 2020, to December 31, 2021; as such, our study could not discuss this correlation. Giani et al. (10) found that in China and Europe, reducing air pollution throughout the COVID-19 pandemic improved long-term health. Wolhuter et al. also observed that the COVID-19 pandemic was effective in reducing air pollutant levels, thereby causing a potential reduction in cardiovascular disease occurrences (11). These findings are comparable to the results obtained in this study. Our study has several strengths. First, all participating patients were diagnosed with acute STEMI on the basis of medical records, abnormal electrocardiogram, and elevated myocardial enzyme levels, and they were confirmed via cardiac catheterization. All the patients who were diagnosed with ADHF had abnormal physical examination results, abnormal echocardiography findings, and elevated NT-ProBNP levels. To our knowledge, there were no problems with diagnosis coding errors. Second, by tracking medical records, it was determined that all patients had lived in the study area for more than 1 year. Third, in this study, the effects of air pollution exposure on acute STEMI and ADHF in the same region were compared before and after the COVID-19 pandemic policies were put into place. Similar to other studies, in this study, it was difficult to determine each patient’s air pollution exposure. However, the COVID-19 pandemic resulted in reduced exposure to air pollution, for instance, by emphasizing the wearing of masks and by working indoors. However, only a small amount of data from a single region and a single hospital were used in this study, which indicates the need for further research to strengthen this study’s credibility. Moreover, as the number of COVID-19 infections increases, the number of people with long-haul COVID symptoms also increases. Future study should be able to explore and study the air pollution-related heart diseases among people with long-haul COVID symptoms.

## 5 Conclusion

The COVID-19 pandemic reduction in PM_2.5_ and PM_10_ also resulted in a decrease in heart attack hospital admissions in Taiwan. COVID-19 pandemic policies may have paradoxically reduced air pollution severity and exposure, which may have led to a decline in cardiovascular events. The governments should continue to formulate better policies to improve air quality to improve citizens’ health.

## Data availability statement

The original contributions presented in the study are included in the article/supplementary material, further inquiries can be directed to the corresponding authors.

## Ethics statement

The studies involving humans were approved by this study was approved by the Institutional Review Board of Tri-Service General Hospital, and all procedures were in accordance with prevailing ethical principles (TSGHIRB No. A202205071). The studies were conducted in accordance with the local legislation and institutional requirements. The ethics committee/institutional review board waived the requirement of written informed consent for participation from the participants or the participants’ legal guardians/next of kin because the informed consent waiver was granted by the Institutional Review Board of Tri-Service General Hospital (TSGHIRB No. A202205071).

## Author contributions

C-CY: Conceptualization, Data curation, Formal analysis, Investigation, Methodology, Resources, Software, Supervision, Validation, Visualization, Writing – original draft, Writing – review & editing. P-JH: Conceptualization, Data curation, Formal analysis, Funding acquisition, Investigation, Methodology, Project administration, Resources, Supervision, Validation, Visualization, Writing – original draft, Writing – review & editing. C-MC: Formal analysis, Methodology, Software, Supervision, Validation, Visualization, Writing – review & editing. P-LC: Conceptualization, Data curation, Formal analysis, Investigation, Methodology, Project administration, Resources, Software, Validation, Writing – review & editing.

## Footnotes

^1^ https://airtw.moenv.gov.tw
